# Towards a global cancer knowledge network: dissecting the current international cancer genomic sequencing landscape

**DOI:** 10.1093/annonc/mdx037

**Published:** 2017-02-03

**Authors:** D. J. Vis, J. Lewin, R. G. Liao, M. Mao, F. Andre, R. L. Ward, F. Calvo, B. T. Teh, A. A. Camargo, B. M. Knoppers, C. L. Sawyers, L. F. A. Wessels, M. Lawler, L. L. Siu, E. Voest, on behalf of the Clinical Working Group of the Global Alliance for Genomics and Health

**Affiliations:** 1 Netherlands Cancer Institute, Amsterdam, The Netherlands; 2 Princess Margaret Cancer Centre, Toronto, Canada; 3 Global Alliance for Genomics and Health, Broad Institute, Cambridge, USA; 4 Yonsei Cancer Research Institute, Yonsei University College of Medicine, Seoul, South Korea; 5 INSERM U981, Université Paris Sud, Institut Gustave Roussy, Villejuif, France; 6 Research, University of Queensland, Brisbane, Australia; 7 Cancer Core Europe, Gustave Roussy, Villejuif, France; 8 National Cancer Centre, Singapore; 9 Oncology, Hospital Sírio Libanês, São Paulo, Brazil; 10 Centre of Genomics and Policy, McGill University, Montreal, Canada; 11 Memorial Sloan Kettering Cancer Centre, New York, USA; 12 Department of Bioinformatics & Statistics, Delft University of Technology, Delft, The Netherlands; 13 Centre for Cancer Research and Cell Biology, Queen’s University Belfast, Belfast, UK

**Keywords:** genomics, molecular profiling, cancer, survey, data sharing

## Abstract

**Background:**

While next generation sequencing has enhanced our understanding of the biological basis of malignancy, current knowledge on global practices for sequencing cancer samples is limited. To address this deficiency, we developed a survey to provide a snapshot of current sequencing activities globally, identify barriers to data sharing and use this information to develop sustainable solutions for the cancer research community.

**Methods:**

A multi-item survey was conducted assessing demographics, clinical data collection, genomic platforms, privacy/ethics concerns, funding sources and data sharing barriers for sequencing initiatives globally. Additionally, respondents were asked as to provide the primary intent of their initiative (clinical diagnostic, research or combination).

**Results:**

Of 107 initiatives invited to participate, 59 responded (response rate = 55%). Whole exome sequencing (*P *=* *0.03) and whole genome sequencing (*P = *0.01) were utilized less frequently in clinical diagnostic than in research initiatives. Procedures to identify cancer-specific variants were heterogeneous, with bioinformatics pipelines employing different mutation calling/variant annotation algorithms. Measurement of treatment efficacy varied amongst initiatives, with time on treatment (57%) and RECIST (53%) being the most common; however, other parameters were also employed. Whilst 72% of initiatives indicated data sharing, its scope varied, with a number of restrictions in place (e.g. transfer of raw data). The largest perceived barriers to data harmonization were the lack of financial support (*P < *0.01) and bioinformatics concerns (e.g. lack of interoperability) (*P = *0.02). Capturing clinical data was more likely to be perceived as a barrier to data sharing by larger initiatives than by smaller initiatives (*P = *0.01).

**Conclusions:**

These results identify the main barriers, as perceived by the cancer sequencing community, to effective sharing of cancer genomic and clinical data. They highlight the need for greater harmonization of technical, ethical and data capture processes in cancer sample sequencing worldwide, in order to support effective and responsible data sharing for the benefit of patients.

## Introduction

In the emerging era of precision medicine, genomic analysis has become an integral component of the diagnostic work-up of cancer patients. Where initially DNA sequencing approaches tested individual cancer ‘hotspot’ loci (e.g. *KRAS* mutational status in colorectal cancer; *EGFR* mutational status in lung cancer), a more precise understanding of the biological basis of malignancy subsequently led to identification and deployment of specific ‘cancer gene panels’ as prognostic or treatment prediction tools. Additionally, the increased interrogative capacity afforded by next generation sequencing (NGS), allied to its decreasing cost, has empowered many institutions worldwide to perform whole exome sequencing (WES) or whole genome sequencing (WGS) on significant numbers of tumour samples. Primary data outputs from these initiatives are increasing exponentially, thus challenging scientific and clinical communities to develop workable solutions for optimal analysis, usage and storage of these datasets. Further complexity is introduced by the need to integrate this genomic data with associated clinical information.

Previously, on behalf of the Clinical Working Group of the Global Alliance for Genomics and Health (GA4GH) (a coalition of researchers, clinicians, patient advocates and life sciences/information technology industries dedicated to implementing worldwide data sharing solutions), we have highlighted the data challenges in cancer genomics [1], emphasized the currently siloed nature of the clinical, pathological and genomic datasets and proposed a blueprint solution that is predicated on a culture of responsible data sharing [2]. However, there is a lack of collective intelligence on current practices in cancer clinical sample sequencing initiatives worldwide. There is a paucity of information on the types of technical NGS platforms/parameters employed and choice of bioinformatics algorithms for analysis. Uniform approaches for collecting matched clinical and-genomic data on outcomes and treatment toxicities are lacking [3]. Information is limited on both institutional enthusiasm for sharing their data and the technical ability to facilitate a data sharing culture. Costs and resources required to establish multi-institutional/international data sharing programs are considerable. From ethical and legal perspectives, data protection legislation/privacy concerns are also challenging, particularly as they can vary significantly according to geographic region [4]. These issues pose significant challenges for effective data harmonization and sharing. Thus, a detailed assessment of the current global cancer clinical sample sequencing landscape is required to inform and enhance present and future data sharing efforts.

Recognizing these information deficits, we performed an international survey of cancer clinical sample sequencing initiatives. This survey was designed to provide an informative snapshot of current activities worldwide and identify potential barriers that may limit data sharing activities, thus informing creation of a global informatics ecosystem that facilitates the sharing of clinical and genomic cancer data at scale.

## Methods

### Recruitment of respondents, survey development and data collection

Methodology for respondent recruitment, survey development and data collection is outlined in the supplementary Appendices S1 and S2, available at *Annals of Oncology* online.

### Statistical analysis

Data collected from *Google Forms* were exported to the *R* statistical package for analysis. Descriptive statistics were used to summarize survey responses. All analyses were performed using *χ*^2^ testing unless otherwise indicated. Likert scales were used to capture the extent of perceived barriers to data sharing (1 = minor barrier, 6 = major barrier). Given that not all questions were mandatory, sample size varied according to the particular question; thus responses have been displayed with the numerator (*n*) and denominator (*N*) (largest possible number of available responses). The denominator is reported for each section once, unless it changes.

## Results

The survey collected responses from July to October 2015. Out of the 107 initiatives invited, 59 responses were received (response rate = 55%). Of the non-responders, 9 initiatives indicated that their activities did not match the survey’s scope or had already been captured in our survey, thus giving a true response rate of 60%. Of the remaining non-responders, the majority resided in the US (*n *=* *23) and Australia (*n *=* *8). None of the Chinese initiatives responded (*n *=* *3). Survey completion rates varied across sections, ranging from 81% [Privacy and Ethics (*n *=* *48, *N* = 59)] to 88% [Barriers (*n *=* *52, *N* = 59)]. Completed surveys included respondents from diverse locations and initiative size, with the majority coming from North America and Europe (supplementary Tables S1, S3 and Figure S1, available at *Annals of Oncology* online). The primary intention of the initiatives were: research [37% (*n *=* *22)], clinical diagnostic [15% (*n *=* *9)], combination [34% (*n *=* *20)] and unknown [14% (*n *=* *8)] as self-nominated by the individual initiative (supplementary Table S1, available at *Annals of Oncology* online). Relevant inter-institutional initiatives in Eastern Europe, Africa or India were not identified.

### Sequencing

#### Platforms

Wide variation in the type of sequencing platforms employed was observed. WES was the most frequently used (*n *=* *28, *N* = 51, 55%) while WGS was also employed in a high number of initiatives (*n *=* *22, 43%). A total of 35% (*n *=* *18) used both WES/WGS, whereas 37% (*n *=* *19) employed neither platform.

Gene-panels were frequently used, with 55% and 51% of responding initiatives indicating that they employ a gene-panel with 51–250 genes (*n *=* *28) or 251–1000 genes (*n *=* *26), respectively. Gene-panels of fewer than 50 genes were also commonly utilized (*n *=* *23, 45%). Very large gene-panels (1001–5000 genes) were used rarely (*n *=* *7, 14%) (Table 1). WES/WGS was employed less frequently in clinical diagnostic initiatives compared with research initiatives (WES: *n *=* *2, *N* = 9, *P *=* *0.03; WGS *n *=* *0, *N* = 9, *P < *0.01). RNAseq/other transcriptomics techniques were employed in 59% (*n *=* *30) and 41% (*n *=* *21), of initiatives, respectively. The use of germline sequencing (as a filter to distinguish somatic from germline single nucleotide polymorphisms) was included in 59% of initiatives (*n *=* *30, *N* = 51). Its use was associated with sequencing intent (*P = *0.02), with only 22% of the Clinical Diagnostics using it reflecting their increased use of small (hotspot) panels.

**Table 1:** Responses to the technical aspects of the survey

		Sequencing platforms					
		*n*	%	Diagnostic *N* = 9	Research *N* = 22	Diagnostic/research, *N* = 20	*P*-value^a^
Panel size (genes)							
Small	< 50	23	45	6 (67%)	7 (32%)	10 (50%)	0.17
Medium	51–250	28	55	5 (56%)	14 (64%)	9 (45%)	0.48
Large	251–1000	26	51	4 (44%)	11 (50%)	11 (55%)	0.86
Very large	1001–5000	7	14	0 (0%)	4 (18%)	3 (15%)	0.40
WES		28	55	2 (22%)	16 (73%)	10 (50%)	0.03
WGS		22	43	0 (0%)	14 (64%)	8 (40%)	0.01
RNAseq		30	59	3 (33%)	16 (73%)	11 (55%)	0.12
Transcriptomics		21	41	3 (33%)	13 (59%)	5 (25%)	0.07
Sequencing depth							
<25		1	2	0 (0%)	0 (0%)	1 (5%)	0.37
25–50		2	4	0 (0%)	0 (0%)	2 (10%)	0.15
51–100		15	29	0 (0%)	7 (32%)	8 (40%)	0.02
101–250		10	20	0 (0%)	5 (23%)	5 (25%)	0.08
251–1000		20	39	9 (100%)	8 (36%)	3 (15%)	0.21
>1000		3	6	0 (0%)	2 (9%)	1 (5%)	0.37
Certification							
ISO		11	22	2 (22%)	4 (18%)	5 (25%)	0.53
CLIA		18	35	5 (56%)	5 (23%)	8 (40%)	0.61
NEN/similar		15^b^	27	4 (44%)	5 (23%)	5 (25%)	0.93
None		20^b^	39	1 (5%)	12 (60%)	6 (30%)	0.01

a *P*-value represents *χ*^2^ testing comparisons between the intent of the particular initiatives.

b One initiative did not indicate their intent.

ISO, international organization for standardization; CLIA, clinical laboratory improvement amendments; NEN, Netherlands Standardization Institute.

#### Sequencing read depth

Questions concerning sequencing read depth employed were completed by 86% of initiatives (*n *=* *51, *N* = 59) (Table 1). Median reported tumour-sequencing depth was 101–250×, with one initiative (2%) indicating depths lower than 25×, whilst three initiatives (6%) indicated depths greater than 1000×. Clinical diagnostic initiatives (*n *=* *9) all reported use of greater sequencing read depth (between 251 and 1000×) compared with research-based initiatives (*P = *0.01). Of the research initiatives, 10 reported read depths greater than 251 (*n *=* *10, *N* = 22, 46%), while four of the combined initiatives reported such depth (*n *=* *4, *N* = 20, 20%).

#### Nucleic acid and protein extraction

The majority of initiatives (*n *=* *29, *N* = 52, 56%) performed sequencing analysis from formalin fixed paraffin embedded (FFPE) and fresh frozen (FF) samples, whereas 27% (*n *=* *14) only employed FFPE as source material and 17% (*n *=* *9) only tested FF samples. Of the initiatives performing the extractions in-house (*N* = 44), the majority indicated extraction of DNA and RNA from the same sample (*n *=* *34, 77%); protein extraction was relatively uncommon (*n *=* *8, 18%).

#### Laboratory certification/accreditation

The majority of Clinical Diagnostic initiatives (95%) held laboratory certification/accreditation compared with research (40%) and combination (70%) initiatives (*P = *0.01) (Table 1). The most common certification was Clinical Laboratory Improvement Amendments (CLIA) (*n *=* *18, *N* = 51, 35%).

### Bioinformatics tools

#### Mutation calling

**Figure 1.**
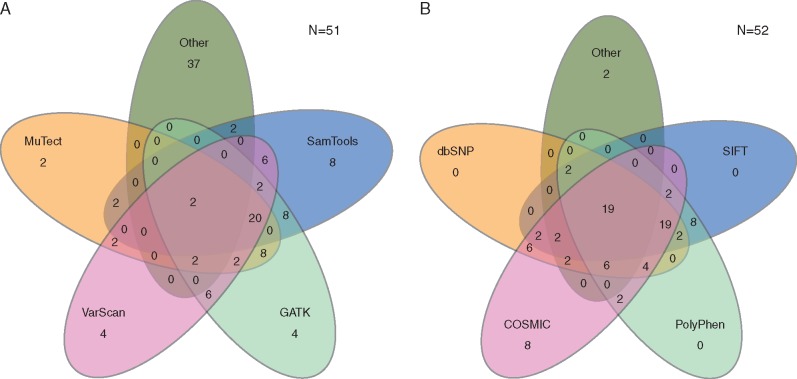
Venn diagrams demonstrating the frequency of bioinformatics pipelines used either in isolation or in combination. Representative images of mutations callers (A) and variant annotation (B) reveal significant heterogeneity. Numbers are expressed as percentages.

The most commonly reported bioinformatics tools were *GATK* (*n *=* *27, *N* = 51, 53%), *Samtools* (*n *=* *25, 49%), *VarScan2* (*n *=* *23, 46%), and *Mutect* (*n *=* *20, 39%) (supplementary Appendix S3, available at *Annals of Oncology* online). The frequency with which these tools were employed is shown in Figure 1A. Logistic regression analysis addressing the type of data employed (prospective, retrospective, combination), indicated that *GATK* is less likely to be used in a prospective study (*P = *0.02). No other significant relationships concerning data type, geographic location or size were identified.

#### Variant annotation

Variant annotation was most commonly performed using *COSMIC* (*n *=* *37, *N* = 52, 71%). *PolyPhen2* (*n *=* *33, 63%), *dbSNP* (*n *=* *33, 63%), and *SIFT* (*n *=* *29, 56%) were also frequently used (supplementary Appendix S3, available at *Annals of Oncology* online). The frequency at which these methods were used (in isolation or in combination) is shown in Figure 1B.

#### Copy number alterations

Of the respondents, 85% (*n *=* *44, *N* = 52) indicated that they estimated copy number alterations (CNAs) from their sequencing data, while 10% indicated not doing so. One initiative reported inference of CNA from targeted panel data.

#### Versioning of pipelines

The majority of initiatives indicated keeping records of which version of their computational procedures (also referred to as software pipelines) to analyse sequencing data that they employed (*n *=* *44, *N* = 52, 85%). Seven initiatives (13%) indicated that they were unsure as to whether the versions of their pipelines were tracked and one initiative (2%) did not track pipeline versions.

### Clinical parameters

#### Merged clinical and genomic data

Of responding initiatives, 47 of 51 (92%, *P < *0.01) attempted to link clinical information to genomic data. No differences in the initiatives’ intent (clinical diagnostic versus research versus combination) and linking of clinical and genomic data were identified (supplementary Table S3, available at *Annals of Oncology* online). Data extraction exclusively employing manual extraction of records was most commonly utilized (*n *=* *23, 45%). Direct deposition of electronic health records (*n *=* *9, 18%, versus manual extraction *P = *0.01), a combination of manual and direct deposition (*n *=* *9, 18%, versus manual extraction *P = *0.01) and other approaches (e.g. in-house direct hospital data feeds) were less frequently utilized (*n *=* *10, 20%, versus manual extraction *P = *0.02) (supplementary Table S3, available at *Annals of Oncology* online). The majority of initiatives used a customized case report form for data collection (*n *=* *34, *N* = 47, 72%).

#### Genotype-drug matching

Of the 51 responding initiatives, 39 (76%) were undertaking genomic-based patient-drug matching for subsequent clinical intervention. Of these, 77% were opportunistically matched (*n *=* *30, *N* = 39) (e.g., phase I studies, off-label use). Nine (23%) initiatives reported that they performed clinical sample sequencing as part of biomarker-driven clinical trials. For measuring treatment efficacy, almost half of responding initiatives used combinations of efficacy endpoints (*n *=* *25, *N* = 51, 49%). The most common endpoints were time on treatment (*n *=* *29, 57%) and response evaluation criteria in solid tumours (RECIST) (*n *=* *27, 53%); however, other parameters such as clinical assessment (*n *=* *14, 27%) were also utilized. Toxicity data were collected in the majority of responding initiatives (*n *=* *28, *N* = 48, 59%).

### Privacy and ethics

Written consent was obtained in 34 initiatives (*N* = 48, 71%), seven initiatives had implied consent/consent waivers (15%). The majority (*n *=* *36, 75%) of initiatives allowed re-contacting of patients for follow-up information. A protocol for communicating somatic genetic results was in place in the majority of initiatives (*n *=* *32, 67%) and a trend to association with the initiative’s intent (clinical diagnostic, research or combination) was identified (*P = *0.06).

A policy for incidental germline findings was in place in 23 of initiatives (48%), but no association was identified with the initiative’s intent (*P = *0.55).

### Data warehousing

Most of the respondents (*n *=* *43, *N* = 50, 86%) indicated that their data storage/warehouse was centralized, for mutation data (*n *=* *47, 94%), copy number alteration estimates (*n *=* *45, 90%), clinical data (*n *=* *42, 84%) and sequencing (BAM) files (*n *=* *41, 82%). Histological data was the least likely to be stored centrally (*n *=* *38, 76%).

### Data sharing

The majority of respondents (*n *=* *36, *N* = 50, 72%, *P < *0.01) indicated that they allow sharing of their data (supplementary Figure S2, available at *Annals of Oncology* online). Fourteen percent indicated not intending to share, while another 14% indicated that they are in the process of developing data sharing policies. No association was identified between data sharing and purpose of the initiative (*P = *0.14). Data sharing typically came with a varied set of restrictions such as regional legislation (e.g. European data that cannot leave the Eurozone, intellectual property (IP) concerns and material transfer agreement restrictions). Certain initiatives remarked that there were significant limitations in transferring raw data between institutions.

### Perceived barriers

The greatest barriers identified (defined as responses >4 on the Likert scale, *N* = 52) were: financial support for data sharing (77%, *P < *0.01), bioinformatics concerns such as lack of conformity and interoperability of bioinformatics pipelines (69%, *P = *0.02), and clinical data capture (60%, *P = *0.19) (supplementary Figure S2, available at *Annals of Oncology* online). Initiatives with 1000 or more patients were more likely to perceive clinical data capture as a barrier compared with smaller initiatives (*P < *0.01). Lack of expertise in the context of rapidly evolving technology (50%) and legal issues (37%) were also raised as potential barriers, whereas privacy/ethics issues (35%) and international legislation (33%) were not considered significant barriers. Of note, bioinformatics and financial concerns did not differ between size of initiative or whether the initiative was clinical diagnostic, research or combination. The free-text commentary of perceived barriers is shown in supplementary Table S4, available at *Annals of Oncology* online.

### Funding

The most frequent source of funding was governmental (*n *=* *11, *N* = 50, 22%), charities (*n *=* *9, 18%), government combined with academic/professional (e.g. AACR/ASCO) sources or charities (*n *=* *5, *N* = 50, 10%), industry (*n *=* *4, 8%), charities combined with industry (*n *=* *3, 6%) and academic/professional societies (*n *=* *2, 4%). The remainder (*n *=* *16, 32%) consisted of hybrid combinations with a frequency of one. Concerns were expressed in relation to funding of international data sharing initiatives (supplementary Table S4, available at *Annals of Oncology* online).

## Discussion

Molecular technologies such as NGS have revolutionized cancer biology discovery. Their successful clinical application depends on the sequencing platform and its robustness, the associated bioinformatics pipeline(s), and the availability of clinically annotated data from patients undergoing therapeutic interventions. Linking clinical and genomic data can justify molecular stratification of patients to specific interventions, but there is a realization that matched data must be available from sufficient numbers of patients to allow statistically robust, clinically meaningful conclusions to be drawn. Collaborative sharing of this information between initiatives increases the value and relevance of the data, for the scientist, the pharmaceutical industry, the clinician, the payer (insurance/taxpayer) and ultimately for the patient. However, effective data sharing is challenging, from technical, clinical, ethical, logistical and regulatory perspectives.

From a technical perspective, respondents to the survey employed a number of sequencing platforms and methodologies. Of these platforms, WES (*P = *0.03) and WGS (*P < *0.01) were more relevant to research application, with low adoption rates in clinical diagnostic initiatives. Conversely, clinical diagnostic initiatives employed greater sequencing depths than research initiatives (*P = *0.012). Surprisingly, nearly 40% of initiatives surveyed did not have clinical molecular diagnostic laboratory certification/accreditation, highlighting a deficiency that must be addressed in order for NGS to be routinely incorporated into mainstream clinical diagnostics.

A key finding was the heterogeneity in variant/mutation calling and variant-annotation tools. Use of a single variant caller was rare and tended to involve products from the sequencing vendor or bespoke in-house algorithms. However, the employment of a suite of variant callers was the preferred approach. This heterogeneity in pipelines compromises the ability to compare results between different clinical sample sequencing initiatives [5]. Efforts to address this lack of harmonization are ongoing, through initiatives such as NCI’s Genome Data Commons [6] and the Somatic Mutation Calling Challenge (SMCC) [7]. The recent development of the GA4GH Application Programming Interface (API) [8] provides an easy-to-use web-based algorithm for improved identification of mutations and rearrangements in sequencing data, and is gaining traction in the translational bioinformatics community.

Over 90% of respondents indicated that they had mechanisms in place to capture linked clinical and genomic data. However, uniformity was lacking for the collection and aggregation of this information. While the majority of institutes employed electronic case report forms, nearly half of the initiatives surveyed were manually extracting clinical data. In order to address this, initiatives such as the ASCO’s CancerLinQ project are developing custom-built electronic feeds from community oncology practices to maximize collection of clinical data [9].

A second challenge relates to the quality of the clinical data collected. Incomplete data sets reduce the value of the information collected, while lack of a cancer specific ontology compromises the ability to aggregate and compare clinical and genomic data from different sources. Building a cancer specific Human Phenotype Ontology (which has been an invaluable asset to the rare diseases community) [10], would significantly enhance phenotype–genotype correlations in the study of malignancy.

This survey also highlighted that longitudinal outcome/toxicity data are not captured through a standardized approach outside of clinical trials. Facilitating routine access to these data (e.g. through development of a minimum dataset) is necessary, in order to maximize the collective learning that can be achieved by aggregating clinical/genomic data, especially when analysing rare variants. It was encouraging that 75% of initiatives indicated that their protocol included the permission to re-contact patients, emphasizing the importance that clinical cancer sample sequencing initiatives place on the capture of follow-up patient outcome and toxicity data.

Over 70% of initiatives were in favor of sharing clinical and genomic data. However, a more detailed evaluation of both quantitative and qualitative responses revealed a number of barriers that exist and must be addressed. Lack of dedicated funding was perceived as the most significant barrier to data sharing activities. Collection of even a minimum clinical dataset has major human and technical resource requirements, leading to significant costs. Dedicated funding streams that actively promote data sharing should be encouraged. In this regard, the recent launch of the Innovative Medicines Initiative *Big Data for Better Outcomes* [11] incentivizes both the scientific and pharmaceutical communities to work together in large-scale data sharing activities. The second most commonly highlighted perceived barrier was lack of interoperability of bioinformatics pipelines, and we have already highlighted how initiatives/activities such as GDC [6], SMCC [7] and the GA4GH API [8] are addressing this challenge.

Issues with consent and data privacy were also raised in the free text narrative, with concerns relating to data protection legislation barriers in particular regions e.g. Europe, and harmonization of consent procedures. It is hoped that the recent decision of the European Commission on the EU-US Privacy Shield will help address inter-continental data privacy issues [12]. These regulatory challenges limit the effectiveness of global cancer knowledge networking. From an ethics perspective, ethics harmonization has been a key theme of GA4GH’s *Framework for Responsible Sharing of Genomic and Health-Related Data* [13] that we suggest should serve as an overarching ethical framework for clinical and genomic data sharing. Additionally, introduction of a federated data sharing approach, where data does not leave the particular legal jurisdiction but can be mined efficiently *in situ*, represents a potential solution for regions that are sensitive to primary data transfer. Concerns were also raised in relation to how data sharing might adversely affect publications and IP issues. Improving the quality of publications through effective data sharing, and developing micro-attribution based rewards where the work of data providers is acknowledged [14] should help allay these fears.

The benefits of data sharing become increasingly relevant as we collectively realize that our current catalogue of actionable cancer mutations is limited, and even there, consensus is lacking. Molecular stratification approaches have identified distinct disease subtypes, some of which may be relatively rare. Thus, a collective approach employing information from data repositories worldwide is increasingly required to identify/verify relevant mutations that can inform improved diagnosis or identify novel targets. Such an approach has already been employed by GA4GH in the BRCA challenge [15], which convened BRCA experts from around the world to work together to share BRCA variants publicly, thus allowing expert review of variant interpretations to determine the pathogenicity of an increased number of variants in the *BRCA1/BRCA2* genes. This work has resulted in BRCA exchange [16], a curated web portal that allows the BRCA community to query the current evidence of any *BRCA1/2* variant present in the aggregated dataset. Extending the BRCA Challenge approach to other genes and cancers would allow a more granular understanding of variant relevance, thereby informing clinical actionability.

We acknowledge that this study has several limitations. By its nature, it is a snapshot at a particular moment in time, in a rapidly advancing field. While our aspiration was to capture responses from cancer sample sequencing initiatives worldwide, there is an enrichment towards initiatives in North America and Europe, due to a combination of an inability to identify cancer clinical sample sequencing collaborative initiatives and/or a lack of response from such initiatives in particular countries/regions (e.g. India, China). Nonetheless, this survey is a first attempt to catalogue cancer clinical sample sequencing activity worldwide and represents a useful benchmark to inform cancer data sharing activities going forward.

### Conclusions

This is the first comprehensive global survey of cancer clinical sample sequencing initiatives. It provides an evidence-based perspective informed by responses from experts worldwide concerning the key barriers to data sharing. It emphasizes the need to break down individual data silos and underscores the requirement to provide robust approaches for clinical and genomic data collection and analysis. It highlights how limited dedicated funding, a dearth of standardized methodologies and a lack of thoughtful integration are hampering clinically relevant data sharing efforts. Developing a bioinformatics ecosystem that delivers open source interoperable solutions to overcome the barriers we highlight, would maximize the potential for responsible but effective sharing of clinical and genomic data for the benefit of cancer patients globally.

## Supplementary Material

Supplementary Data
