# Supplementary material for: Towards a global cancer knowledge network: dissecting the current international cancer genomic sequencing landscape
Source: Ann Oncol. 2017 Feb 3;28(5):1145–51. doi: 10.1093/annonc/mdx037 (PMC5406763; doi:10.1093/annonc/mdx037)
Supplement: Supplementary Data [file mdx037_supp.zip › Supplementary Information clean version.docx]

**Supplementary Information**

**Appendix S1 Recruitment of Respondents**

International cancer sequencing initiatives were identified through the GA4GH membership network and cross checked with registrations at previous GA4GH meetings (London (UK), San Diego (USA), Leiden (NL)), with an emphasized preference for the participation of initiatives that involve inter-institution collaborations. Specific contacts were identified to enhance recruitment from different geographical regions (Australia; Asia; Europe; South America; North America). Participants were invited to complete a web-based survey (Google Forms, Alphabet Inc., USA) and consent was assumed based on returned response. Respondents were invited by email in July 2015 to participate and email reminders were sent to non-responders. Invitees who did not complete the survey within 2 weeks were sent further reminders, either directly or via our professional network, to ensure their participation. This process was repeated until survey closure in October 2015.

## Appendix S2 Survey development and data collection

A multi-item survey was designed to assess six information domains associated with cancer sample sequencing initiatives. The complete survey is provided as Supplemental Appendix 1. The 86 questions were distributed as follows: 1) demographics (e.g., location of initiative, objective, regional scope, type of data, samples per year analyzed) (12 questions in total); 2) clinical data collection (e.g., whether genomic data are linked to clinical data; methods of data extraction; methods for target drug matching and if so, treatment efficacy and toxicity assessments) (15 questions); 3) nature and scope of genomic platforms (e.g., platform employed for clinical diagnostic or research use, type of bioinformatics pipelines, choice of data warehousing) (33 questions); 4) privacy and ethical considerations (e.g., informed consent, data protection legislation, procedures for re-contacting patients) (7 questions); 5) funding source (2 questions); 6) perceived barriers to data sharing (17 questions). Respondents were given the opportunity to add additional comments in text boxes to expand on specific details related to their particular initiative. Additionally, given the multidimensional nature of the survey, respondents were given the opportunity to save responses and return at multiple time points, thus allowing consultation with internal staff to ensure comprehensive data entry in relation to their specific initiative. Lastly, initiatives were asked to identify whether the purpose of their platform was for clinical decision making (which we have labeled “Clinical Diagnostic), research (“Research”) or a combination of both (“Combination”).

**Appendix S3: Extended References for Mutations Calling and Variant Annotation**

|  | Reference |
| --- | --- |
| GATK | [1] |
| Samtools | [2] |
| VarScan2 | [3] |
| Mutect | [4] |
| COSMIC | [5] |
| Polyphen2 | [6] |
| dbSNP | [7] |
| SIFT | [8] |

**References**

1. McKenna A, Hanna M, Banks E et al. The Genome Analysis Toolkit: a MapReduce framework for analyzing next-generation DNA sequencing data. Genome Res 2010; 20:1297-1303

2. Li H, Handsaker B, Wysoker A et al. The Sequence Alignment/Map format and SAMtools. Bioinformatics 2009; 25:2078-2079

3. Koboldt DC, Zhang Q, Larson DE et al. VarScan 2: somatic mutation and copy number alteration discovery in cancer by exome sequencing. Genome Res 2012; 22:568-576

4. Cibulskis K, Lawrence MS, Carter SL et al. Sensitive detection of somatic point mutations in impure and heterogeneous cancer samples. Nat Biotechnol 2013; 31:213-219

5. Bamford S, Dawson E, Forbes S et al. The COSMIC (Catalogue of Somatic Mutations in Cancer) database and website. Br J Cancer 2004; 91:355-8

6. Adzhubei IA, Schmidt S, Peshkin L et al. A method and server for predicting damaging missense mutations. Nat Methods 2010; 7:248-249

7. Sherry ST, Ward M, Sirotkin K. dbSNP—database for single nucleotide polymorphisms and other classes of minor genetic variation. Genome Res 1999; 9:677-679

8. Ng PC, Henikoff S. SIFT: Predicting amino acid changes that affect protein function. Nucleic Acids Res 2003; 31:3812-3814

**Supplementary Table S1: A list of initiatives/institutions responding to the survey.**

| **Initiatives** | | | |
| --- | --- | --- | --- |
| **Clinical Diagnostic (*n*=9)** | **Research *(n*=22)** | **Combination (*n*=20)** | **Unknown (*n=*8)** |
| AC Camargo Cancer Center (Sth America) | Australian Genomics Health Alliance (Australia) | BRCA challenge/ENIGMA (International) | CANCERLINQ (Nth America) |
| CCI, SCH Zero Childhood Cancer Program (Australia) | Cancer Core Europe (Europe) | Cancer Driver Discovery Program (Nth America) | COTA (Nth America) |
| Individualized Molecular Profiling for Assigning Cancer Therapeutics (Asia) | CANCER 2015 (Australia) | Center for Personalized Cancer Treatment (Europe) | ISPY (Nth America) |
| MD Anderson Precision Oncology Decision Support Core (Nth America) | Center for Personalised and Precision Health (Asia) | Centre for Translational Research and Diagnostics (Asia) | National Cancer Institute (Europe) |
| MSK-IMPACT (Nth America) | Clearinghouse (Nth America) | Clinical Genome Resource (International) | NCI-MATCH (Nth America) |
| NEXT-1 (Asia) | CREATE (Europe) | Clinical Trial Sequencing Program (Nth America) | SHIVA-01 (Europe) |
| SAFIR (Europe) | DKFZ (Europe) | GAIN/iCat (Nth America) | University of Sao Paulo (Sth America) |
| S-CORT (Europe) | EORTC-SPECTA (Europe) | G-DOC (Nth America) | FOCUS-4 (Europe) |
| University of Cambridge (Europe) | ICGC (International) | GENIE (Nth America) |  |
|  | Institut Gustave Roussy (Europe) | Genomics England (Europe) |  |
|  | Korea Actionable Genome Consortium (Asia) | Genomic Initiative of Gastric Cancer Treatment (Asia) |  |
|  | Novartis Institutes for Biomedical Research (International) | Hospital Israelita Albert Einstein (Sth America) |  |
|  | ORIEN (Nth America) | IMPACT/COMPACT (Nth America) |  |
|  | Personalized OncoGenomics (Nth America) | Jewish General Hospital (Nth America) |  |
|  | POLARIS (Asia) | Karolinska Institute (Europe) |  |
|  | SAFIR02 (Europe) | My Pathway (Nth America) |  |
|  | The San Antonio 1000 Cancer Genome Project (Nth America) | Oregon Health & Science University (Nth America) |  |
|  | TAPUR (Nth America) | University of Melbourne (International) |  |
|  | TCGA (International) | University of Tokyo (International) |  |
|  | Treehouse Childhood Cancer Project (International) | Vall d'Hebron (Europe) |  |
|  | University of Colorado (Nth America) |  |  |
|  | WIN consortium (International) |  |  |

CCI, SCH: Children’s Cancer Institute, Sydney Children’s Hospital; COTA: Cancer Outcomes Tracking and Analysis; ENIGMA: Evidence-based Network for the Interpretation of Germline Mutant Alleles; EORTC/SPECTA: Screening Patients for Efficient Clinical Trial Access; GAIN/iCAT: Genomic Assessment Improves Novel Therapy / Individualized Cancer Therapy; GENIE: Genomics, Evidence, Neoplasia, Information, Exchange (AACR); G-DOC: Georgetown Database of Cancer; ICGC: International Cancer Genome Consortium; NCI-MATCH: National Cancer Institute - Molecular Analysis for Therapy Choice; S-CORT: Stratification in colorectal cancer; ORIEN: The Oncology Research Information Exchange Network; POLARIS: Personalized Omic Lattice for Advanced Research and Improving Stratification; TAPUR: Targeted Agent and Profiling Utilization; TCGA: The Cancer Genome Atlas

**Supplementary Table S2:** **Demographic of respondents** including the regional scope of their initiatives, the (expected) annual accrual, and the primary intent of the initiative. A Clinical Diagnostic intent was defined as an analysis that occurred within the context of clinical decision making.

|  | ***N* (%)** |
| --- | --- |
| **Number of initiatives** | 59 |
| **Regional Location**  North America  Europe  Asia  Australia  South America  Intercontinental | 20 (34)  16 (27)  7 (12)  3 (5)  3 (5)  10 (17) |
| **Regional Scope**  Institutional (local)  Multi-institutional (regional) / National  European Union  International  Unknown | 15 (25)  28 (47)  4 (7)  10 (17)  2 (3) |
| **Patient Samples per Year**  1-500  501-5000  >5000  Unknown | 21 (36)  22 (37)  7 (12)  9 (15) |
| **Intent of Analysis**  Clinical Diagnostic  Research  Clinical Diagnostic / Research  Unknown | 9 (15)  22 (37)  20 (34)  8 (14) |

**Supplementary Table S3**: **Mechanisms for data linkage and extraction.**

|  | | **Intent of Initiative** | | | |
| --- | --- | --- | --- | --- | --- |
|  |  | **Diagnostic (*n*=9)** | **Research (*n*=22)** | **Diagnostic / Research (*n*=20)** | **Unknown (*n*=8)** |
| **Clinico-genomic data linkage** | Yes  No  Unknown  Other | 6 (67)  0  1 (11)  2 (22) | 21 (95)  0  1 (5)  0 | 17 (85)  1 (5)  2 (10)  0 | 3 (38)  0  4 (50)  1 (13) |
| **Data extraction** | Direct  Manual  Both  Other  Unknown | 1 (11)  5 (56)  0  1 (11)  2 (22) | 3 (14)  8 (36)  5 (23)  5 (23)  1 (5) | 4 (20)  8 (40)  5 (25)  2 (10)  1 (5) | 1 (13)  2 (25)  0  1 (13)  4 (40) |
| **Use of CRF form** | Yes  No  Unknown  Other | 5 (56)  2(22)  0  2 (22) | 15 (68)  5 (23)  0  2 (9) | 10 (50)  6 (30)  0  4 (20) | 4 (50)  0  0  4 (50) |

CRF: Case report form

**Supplementary Table S4:** Thematic analysis of free text responses from responding initiatives with regards to barriers to data sharing

| **Domain** | **Theme** | **Selected Quotes** |
| --- | --- | --- |
| **Funding** | - Competitive funding - Difficult in obtaining funds for long term commitment - Barriers not limited to application of sequencing by also for data maintenance and collation. | “*Very hard to get support for an international consortium as no single country wants to pay for collation of data/information and lab tests that are going to be done in other countries despite the global benefit*.”  “*This is an expensive endeavor and we need to demonstrate its value before we can secure sustainable funding. As the sequencing costs drop and the numbers increase we can enjoy some economy of scale however the costs of the data analysis are still significant.*”  “*Commercial labs do not invest in developing capacity for digital reporting of molecular test results. This greatly constrains the availability of such data and is a major hurdle.”* |
| **Bioinformatics** | - Improvement needed with bioinformatic support (e.g. variant calling, interpretation of variants, amplification calling) - Investment in infrastructure required - Workforce recruitment and expertise - Unknown when to discard data - Challenge in developing interoperable platforms across institutions | “*We are in the process of recruiting additional bioinformaticians as well as secure further funding. A lot more support [is] required to match the demand of the ongoing / planned projects.*”  “*Data sharing of large scale data is a substantial hurdle as genomic assays differ.*”  “*The distillation of whole DNA and RNA data into useful and comprehensible data packages for each patient is a huge data challenge.*” |
| **Clinical** | - Lack of standardized collecting systems - Variability in completion rates - Difficulty when obtaining clinical data from a variety of sources | “*Collection of clinical data remains the Mt Everest of problems in the genomics world. There is no simple solution and it is therefore very expensive.*”  “*Data from consented patients is derived from many different primary sources and the unstructured nature of the data is a major challenge.*”  “*We know there will be incomplete clinical data; however, we do not yet know the scope of the problem and how much of a difficulty it presents.*” |
| **Lack expertise** | - Expertise lacking with clinicians given rapidly developing technology - Reliance on individual groups to provide expertise | “*Need virtual tumor boards with broad disease and biomarker expertise.*”  “*As the clinical aspects of this data will be used by medical oncologists there is a huge educational gap that will need to be filled in the next few years. Oncologists will have to learn a lot about genomics and how these tests may or may not apply to their patients - this is not taught in medical school.”* |
| **Legal** | - Legislation not adequate for genomic data - Intellectual property agreements are difficult - No unified legal framework | “*It would help to have a unified legal framework or guidelines to govern legal issues.”*  “*IP agreements with partner companies are challenging and take a long time.*” |
| **Privacy** | - No consensus on the differences between private and identifiable data - Significant worldwide variations in definition of privacy | *“There is no consensus on what is considered "private" and "identifiable" information. This creates legal hurdles in developing data sharing agreements.”*  *“Misunderstanding of the real privacy issues greatly diminishes the usability of the data generated. Patients should be given the option to release their data without barriers in their informed consent, if they so wish.”* |
| **International Legislation** | - Significant barriers envisaged with multi-institutional transatlantic collaborations | “*Procedures exist to exchange data with foreign institutions; However, differing legal requirements make this difficult. [This] typically leads to delays of a few months (up to a year) in data exchange.*”  *“New EU regulation for data protection might impose additional constraints.”*  *“Unclear what regulations need to be followed when developing international data sharing agreements.*”  “[International legislation poses a] *major hurdle in the USA, with recent mandate for FDA oversight of the laboratory-based omics technologies in prospective clinical trials.*” |

**Supplementary Figure Legend**

**Figure S1:** Map indicating location of responding initiatives to the survey.

**Figure S2:** Pie-chart demonstrating the majority of responding initiatives are currently sharing data or have plans to share data (Fig S2A). Barriers to data sharing are displayed (Fig S2B) and presented as responses greater than 50% on a Likert scale from 1 (minor barrier) to 7 (major barrier).
