# Supplementary material for: Towards a global cancer knowledge network: dissecting the current international cancer genomic sequencing landscape
Source: Ann Oncol. 2017 Feb 3;28(5):1145–51. doi: 10.1093/annonc/mdx037 (PMC5406763; doi:10.1093/annonc/mdx037)
Supplement: Supplementary Data [file mdx037_supp.zip › Global Genomic and Clinical Data SharingV2.0.pdf]

Edit this form

Answer questions you want to pre-fill, then click submit.

Global Genomic and Clinical Data Sharing

Global Alliance for Genomics and Health; Cancer Task Team - 'Data sharing and harmonization effort'

You have been invited to take part in this survey as we have identified you as a leading individual for a genomics initiative which we have explicitly named in the email. In answering the questions in this survey, please keep in mind that all responses are for this initiative only.

The questions in this survey are grouped by theme. Each theme page has a free text field in which you can enter additional thoughts not covered by the questions. The following topics will be addressed, after the current page in which you can identify yourself. We will ask questions about data collection, the implementation of genomic platforms, data warehousing, privacy and ethics, types of studies, funding, and hurdles.

We realize that the breath of questions in this survey may prompt you to consult your staff members on legal, clinical or technical issues. For this reason, you can decide after completing a page to 'log out' and continue later. You can do so by selecting save/submit in the optional navigate question, and then submitting your responses to the system. You will be send an email containing a link that allows you to resume the survey. You can forward this email to your staff and task them with completing specific pages.

* Required

About you

Please tell us who you are, which institute you represent, and the position you hold. Optionally, you can include your email address for potential followup discussions.

Your name *

Institute *

Position *

Location *

Email address *

Navigate

Optional. We understand that there may be questions for which you need to consult your local expert, so you can save/submit now and continue later or you can choose the next topic in this survey.

- Let me select next topic
- Save/Submit

Navigation page

This page allows you to navigate the survey. Please select a question-theme.

Choose a topic

Please choose a survey topic

- General questions on data collection
- Clinical data collection
- Implementation of genomic platforms
- Data warehousing
- Privacy and Ethics
- Funding
- Hurdles
- Last page/suggestions
- Save/submit

General questions on data collection

The following series of questions will deal with the various data collection initiatives that you have committed to.

Name of the initiative *

Please state the name of the initiative and website, or any published reference

Main objective *

Please state the main objective of the initiative

Regional scope *

Please state what the regional scope of the initiative is; national/international (please name the countries).

Anticipated number of patient samples per year *

Please indicate the expected number of patients accrued in the initiative

Date of initiation *

Please indicate when the initiative started

mm/dd/yyyy

Type of data *

Please indicate whether the collected data is of retrospective, prospective or a mixture of both.

- ○ Retrospective
- ○ Prospective
- ○ Combination of retro/prospective
- ○ Unknown

Number of centers involved *

Additional comments

You can enter additional comments here

Navigate

Optional. We understand that there may be questions for which you need to consult your local expert, so

you can save/submit now or you can choose the next topic in this survey.

- ○ Let me select next topic
- ○ Save/Submit

Clinical data collection

The following set of questions deal with the clinical data gathering.

Genomic data with associated clinical data *

Please indicate whether clinical data is tracked in this initiative

- ○ Yes
- ○ No
- ○ Unknown
- ○ Not structurally

Do you collect clinical data that are linked to genomic data in your initiative? *

Please indicate whether you collect the clinical data of the subjects

- ○ Yes
- ○ No
- ○ Unknown
- ○ Other: ____

How do you extract clinical data for your initiative? *

- ○ Direct deposition from electronic health records
- ○ Manual extraction of data by review of electronic health records
- ○ Other: ____

Do you collect clinical data using a customized Case Report Form (CRF) for your initiative? *

- ○ Yes
- ○ No
- ○ Unknown
- ○ Other: ____

How do you collect outcome data on efficacy of treatment? *

Please indicate whether you collect outcome data on efficacy of treatment (Check all that apply)

- ☐ RECIST (or similar) based
- ☐ Time on treatment
- ☐ Clinical assessment only (not RECIST)
- ☐ Not collected
- ☐ Other: ____

Do you collect toxicity data on treatment? *

- ○ Yes
- ○ No
- ○ Unknown
- ○ Other: ____

Clinical Application of genomic results

The following set of questions inquire about the clinical application of the genomic results you generate in your initiative.

Does your initiative attempt to match patients to clinical trials based on their genotypes identified by genomic sequencing? *

Yes

- Yes
- No
- I do not know
- Other:

If your genomic initiative is not attemping to match patients to clinical trials based on their genotypes, what are the goals and applications of your initiative *

- Not applicable
- Other:

About your largest trial

For the largest clinical trial in your initiative, please provide the following (if answer is not known, please put I don't know):

Name of the trial

Sponsorship (industry sponsored or investigator initiated)

Target sample size for patient enrollment

Please provide enrollment target, not screening target

Does the trial involve investigational agents

- Yes
- No

Does the trial involve approved drugs

- Yes
- No

Please provide the NCT number if known

Additional Comments

Here you can enter additional comments concerning the clinical application of the genomic results you generate in your initiative

Navigate

Optional. We understand that there may be questions for which you need to consult your local expert, so you can save/submit now or you can choose the next topic in this survey.

- Let me select next topic
- Save/Submit

Implementation of genomic platforms

The following questions will deal with how you have implemented the genomic platforms in your workflow.

Sequencing infrastructure *

Please indicate which type of sequencing capacity you employ

- Centralized testing
- Local testing
- Hybrid
- Unknown
- Other:

Sequencing types *

Which of the following sequencing platforms do you regularly employ in your institutite/initiative. Gene panels refer to hot-spot panels or targeted gene panels. WES refers to whole exome sequencing, and WGS to whole genome sequencing. RNAseq refers to RNA sequencing, and Other Transcriptomics to transcription profiling with methods other than RNAseq

	Yes	No	Unknown
Small Gene panels <50			
Medium Gene panels 51 - <250			
Large Gene panels 251 - <1000			
Very large gene panel 1001 - < 5000			
WES			
WGS			
RNAseq			
Other Transcriptomics			

Certifications *

Please indicate which certifications your workflow received. CLIA is Clinical Laboratory Improvement Amendments. ISO is the International Organization for Standardization, and National - for example NEN is The Netherlands Standardization Institute.

	Yes	No	Unknown

CLIA	◯	◯	◯
ISO	◯	◯	◯
National (e.g. NEN)	◯	◯	◯

Germline sequencing included *

Please indicate whether your workflow includes matched germline samples (i.e., normal DNA as match) as a standard operating procedure (SOP)

- ◯ Yes
- ◯ No
- ◯ Unknown

Purpose of the test

Please indicate whether you use the test data for clinical descision making or research, tick which apply

- ☐ Clinical diagnosis
- ☐ Research

Sample quality control

Please indicate whether you have QC measures in place for the tumor percentage, and extraction yields. You may also indicate other QC measures you feel are important.

- ☐ Tumor percentage
- ☐ DNA/RNA extraction yields
- ☐ DNA quality assessment (by PCR)
- ☐ Other: ______

Tumor sequencing depth *

Please indicate the typical average tumor sequencing depth you employ

- ◯ < 25
- ◯ 25 - 50
- ◯ 51 - 100
- ◯ 101 - 250
- ◯ 251 - 1000
- ◯ > 1000

Uniformity in data collection *

Please indicate whether the type of samples sequenced are fresh or FFPE, or that both types are sequenced.

- ◯ Fresh frozen
- ◯ FFPE
- ◯ Both

Combined isolation of DNA/RNA/protein from tumors *

Please indicate whether you have protocols for combined extraction and for which type of sample, tick which apply.

- ☐ DNA
- ☐ RNA
- ☐ Protein
- ☐ Fresh biopsies
- ☐ FFPE

- [ ] Other: ____

Variant calling *

Which tools/pipelines do you use for calling variants (tick that apply)

- [ ] Samtools
- [ ] GATK
- [ ] VarScan
- [ ] MuTect
- [ ] Other: ____

Variant annotation *

How do you annotate/filter your variants for impact

- [ ] SIFT
- [ ] PolyPhen
- [ ] FATHMM
- [ ] COSMIC
- [ ] dbSNP
- [ ] ESP (exome sequencing project)
- [ ] Other: ____

Versioning of pipelines *

Different versions of tools can give (slightly) different resuls. Please indicate whether you track the software versions used for obtaining your results.

- ( ) Yes
- ( ) No
- ( ) Unknown

Copy number alterations *

Please indicate whether you extract CNA from your sequencing data

- ( ) Yes
- ( ) No
- ( ) Unknown

Additional comments

Here you can enter additional comments

Navigate

Optional. We understand that there may be questions for which you need to consult your local expert, so you can save/submit now or you can choose the next topic in this survey.

- ( ) Let me select next topic
- ( ) Save/Submit

Data warehousing

The following series of questions are about your data warehousing strategy.

Centralized storage *

Please indicate whether you have centralized your data storage facilities

- Yes
- No
- Unknown

Centralized data storage *

Please indicate whether you store the sequencing files (BAM), mutation calls, CNA calls, clinical data

	Yes	No	Unknown
Sequence data (BAM)			
Mutation calls			
Copy number alteration calls			
Clinical data			
Histology			

Uniform Unique Identifiers *

Do you have a system in place that ensure each sample receives a unique 'barcode' identifier, and that these are linked to the patient.

- Yes
- No
- Unknown
- Other:

Data Access *

What are your policies concerning accessing the data and enforcing these access control rights

Data Ownership

Please specify which guidlines you've implemented to deal with data ownership

Data sharing *

Does your initiative allow for data sharing outside of your institution

Yes

No

Other:

Additional comments

You can enter additional comments here

Navigate

Optional. We understand that there may be questions for which you need to consult your local expert, so you can save/submit now or you can choose the next topic in this survey.

Let me select next topic

Save/Submit

Privacy and ethics

The data from NGS studies poses interesting challenges as the sequencing results do not only reveal information about the patient, but also about their relatives. Whether the patient wants to be informed of results that are not of primary interest, and whether results that imply differential risk profiles for its relatives can be communicated, pose real ethical challenges. Similarly, the recording and sharing of the NGS and clinical data are subjected to privacy regulations.
We would like to know whether you've considered or implemented the following protocols

Informed consent *

Does your initiative obtain informed consents from patients

Implied consent or consent waiver

Specific written consent

Other:

Recontacting patients *

Can patients be recontacted for follow up information

Yes

No

Other:

Protocol for data ownership *

Does your initiative include a protocol for data ownership

Yes

No

Other:

Protocol for communicating genetic results *

Yes

◯ No

Policy for incidental germline findings *

Do you have a policy to refer incidental finding to clinicial genetics department

◯ Yes

◯ No

Is your initiative for profit *

Please indicate whether any potential revenues from the derived work of the imitative benefits shareholders or the initiative itself

◯ Yes

◯ No

◯ Unknown

◯ Other: ________

Additional comments

Here you can enter additional comments related the privacy and ethics protocols you use

Navigate

Optional. We understand that there may be questions for which you need to consult your local expert, so you can save/submit now or you can choose the next topic in this survey.

◯ Let me select next topic

◯ Save/Submit

Funding

Please share with us how your initiative(s) are funded

Funding source *

Please indicate the type of the largest funding source for your initiative (check two at most)

☐ Government

☐ Charity

☐ Industry

☐ Academic (AACR, ASCO)

☐ Other: ________

Additional comments

You can enter additional comments here

Navigate

Optional. We understand that there may be questions for which you need to consult your local expert, so you can save/submit now or you can choose the next topic in this survey.

- Let me select next topic
- Save/Submit

Hurdles

In this section we inquire about your perception of hurdles in the implementation of the genetic data sharing in your initiative/institute.

Bioinformatics *

Please rate on a scale from 1 to 6 whether you expect bioinformatics to be a hurdle to your efforts

	1	2	3	4	5	6	
Minor	○	○	○	○	○	○	Major

Bioinformatics

You can enter additional comments here

Capturing clinical data *

Please rate on a scale from 1 to 6 whether you expect the clinical data to be incomplete

	1	2	3	4	5	6	
Minor	○	○	○	○	○	○	Major

Incomplete clinical data

You can enter additional comments here

Financial support *

Please rate on a scale from 1 to 6 whether you expect financial support to be a hurdle to your efforts

1 2 3 4 5 6

Minor ○ ○ ○ ○ ○ ○ Major

Financial support

You can enter additional comments here

Legal barriers *

Please rate on a scale from 1 to 6 whether you expect legal barriers to be a hurdle to your efforts. Examples of such barriers are ownership and copyright laws.

1 2 3 4 5 6

Minor ○ ○ ○ ○ ○ ○ Major

Legal barriers

You can enter additional comments here

International legislation *

Please rate on a scale from 1 to 6 whether you expect international legislation to be a hurdle to your efforts. Examples of hurdles posed by international legislation (difference between national legislation) are privacy protection laws that are incompatible with one another.

1 2 3 4 5 6

Minor ○ ○ ○ ○ ○ ○ Major

International legislation

You can enter additional comments here

Privacy or ethics *

Please rate on a scale from 1 to 6 whether you expect privacy or ethics to be a hurdle to your efforts

1 2 3 4 5 6

Minor ○ ○ ○ ○ ○ ○ Major

Privacy or ethics

You can enter additional comments here

Authorships/Rights to publish *

Please rate on a scale from 1 to 6 whether you expect authorship to be a hurdle to your efforts

	1	2	3	4	5	6	
Minor	○	○	○	○	○	○	Major

Lack of expertise in some areas *

Please rate on a scale from 1 to 6 whether you expect the lack of expertise in one or more areas to be a hurdle to your efforts

	1	2	3	4	5	6	
Minor	○	○	○	○	○	○	Major

Lack of experise

You can enter additional comments here

Additional comments

Navigate

Optional. We understand that there may be questions for which you need to consult your local expert, so you can save/submit now or you can choose the next topic in this survey.

○ Let me select next topic

○ Save/Submit

Last page

You have almost finished the survey. We appreciate all your kind input and value this information highly. We may contact you to further elaborate on some aspects addressed here if that is required. On behalf of the authors we thank you once more, and allow for your additional comments in the text box below.

Further comments

Submit

Never submit passwords through Google Forms.

Powered by Google Forms

This content is neither created nor endorsed by Google.

Report Abuse - Terms of Service - Additional Terms
