# Supplementary figures and images for: Towards a global cancer knowledge network: dissecting the current international cancer genomic sequencing landscape
Source: Ann Oncol. 2017 Feb 3;28(5):1145–51. doi: 10.1093/annonc/mdx037 (PMC5406763; doi:10.1093/annonc/mdx037)

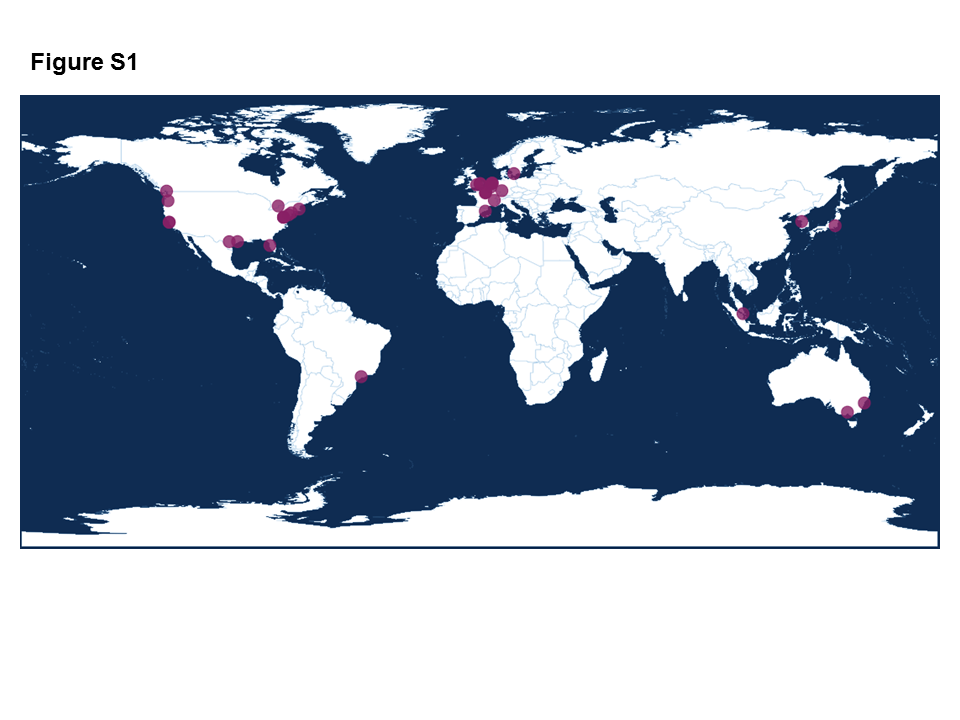

Supplement: Supplementary Data [file mdx037_supp.zip › Figure S1.tif]

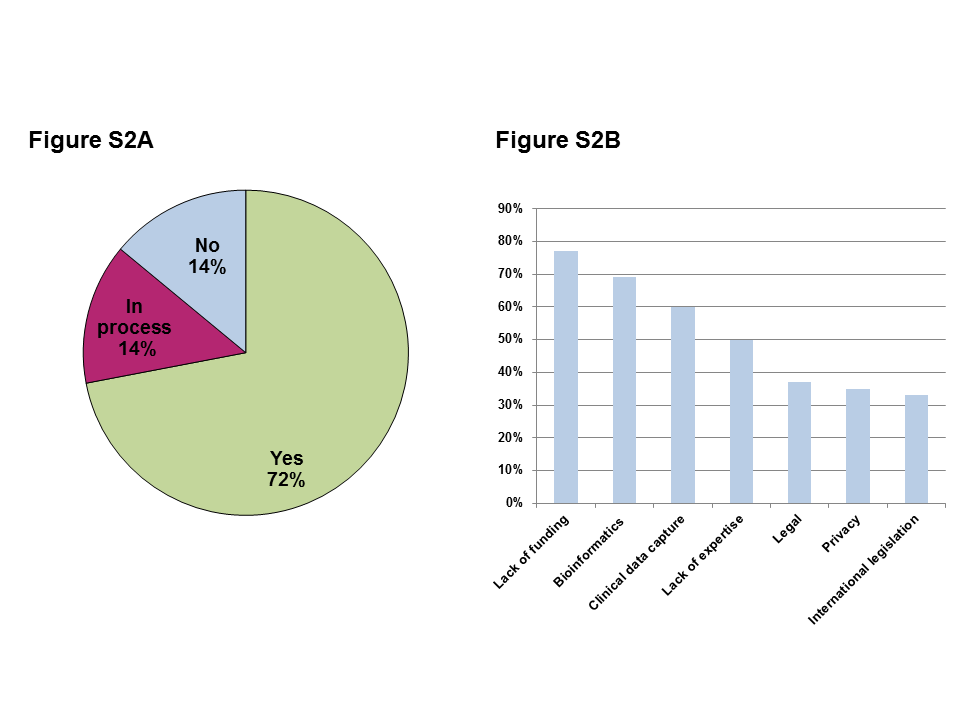

Supplement: Supplementary Data [file mdx037_supp.zip › Figure S2.tif]
